# Structural and genetic analysis of neutralizing antibodies reveals mechanisms of GII.4 norovirus antigenic evolution

**DOI:** 10.21203/rs.3.rs-9077120/v1

**Published:** 2026-03-31

**Authors:** Gabriel Parra, Kelsey Pilewski, Anisa Ghosh, Kentaro Tohma, Lauren Ford-Siltz, Adrian Hernandez, Monica Pajuelo, Michael Landivar, Rebecca DuBois

**Affiliations:** FDA; FDA; University of California Santa Cruz; FDA; FDA; FDA; Universidad Peruana Cayetano Heredia; FDA; University of California, Santa Cruz

**Keywords:** Norovirus, Antigenic evolution, Epitope mapping, Viral evolution

## Abstract

Noroviruses are a leading cause of acute gastroenteritis worldwide, yet no licensed vaccines or antivirals are available. A major barrier to broadly protective vaccine development is the extensive genetic and antigenic diversity of these viruses, leading to immune escape. However, the structural mechanisms underlying this immune escape remain incompletely defined. Here, we analyze a panel of monoclonal antibodies generated against a pandemic GII.4 variant to define the molecular determinants of neutralizing immunity. Guided by immunogenetic features and cross-reactivity patterns spanning four decades of viral evolution, we resolved the atomic structures of two neutralizing antibodies targeting the principal immunodominant antigenic sites, A and G. We show that the spatial positioning of antigenic site G shapes neutralizing responses and that coordinated substitutions within these epitopes drove the rapid antigenic transitions observed between 2004 and 2012.Together, these findings establish a structural framework for GII.4 antigenic evolution and inform rational vaccine design.

## 1. Introduction

Norovirus is a leading cause of acute gastroenteritis across all age groups worldwide. It is a particularly significant public health burden due to its ability to cause large outbreaks in crowded settings such as schools, cruise ships, nursing homes, and healthcare facilities. The disease is characterized by the sudden onset of vomiting and diarrhea, typically lasting 1–3 days. While usually self-limiting, norovirus can lead to severe dehydration and hospitalization, especially in young children, the elderly, and immunocompromised individuals. As a result, norovirus has been associated with approximately 200,000 annual deaths globally and contributes to substantial healthcare costs^1, 2, 3^.

The norovirus virion features an icosahedral capsid composed of two structural proteins: the major capsid protein (VP1) and the minor capsid protein (VP2). During infection, the protective host immune response predominantly targets VP1^4, 5, 6^, making it the central component of all current vaccine strategies^7, 8^. A significant obstacle in developing an effective vaccine is the extensive genetic and antigenic diversity of the virus. Among the various genotypes that can infect humans, the GII.4 genotype is responsible for more than 50% of global norovirus infections^9, 10, 11^. Its dominance has been attributed to the continual emergence of antigenically distinct variants that can evade the immune response elicited from prior infections^12, 13, 14, 15^.

**Figure 1:**
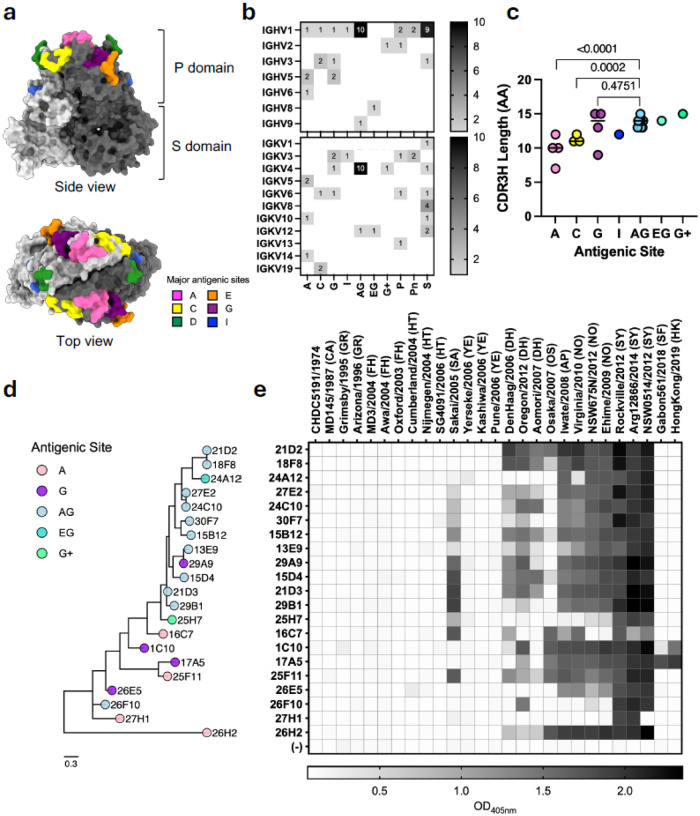
Immunogenetics and cross-reactivity patterns of mouse monoclonal antibodies (mAbs) developed against the pandemic GII.4 Sydney 2012 variant. **a,** Structural model of the GII.4 Sydney 2012 capsid protein (VP1) dimer, with major variable antigenic sites highlighted. VP1 monomers are shown in light and dark gray. The structural model of the VP1 dimer (Protein Database [PDB]: 7K6V) was rendered using UCSF ChimeraX^50^. **b,** Frequency of heavy- and light-chain variable gene usage by antigenic site. P and S denote undefined conserved sites on the P domain and S domain. Pn denotes neutralizing antibodies mapped on the conserved site on the P domain. **c,** CDRH3 length of antibodies recognizing each antigenic site, as determined by IMGT reference alignment^39^. P values of two-tailed unpaired t test are provided. **d,** Phylogenetic relationships among heavy-chain CDR3 amino acid sequences of antibodies recognizing antigenic sites A, G, both (AG), or G and other sites (EG, G+). **e,** Reactivity of each mAb (10 μg/mL) against a panel of 27 VLPs (0.5 μg/mL) representing GII.4 noroviruses circulating between 1974 and 2019. The heatmap presents average OD_405nm_ values from duplicate wells. Names of the GII.4 variants are abbreviated and indicated within parentheses as follows: Camberwell 1987 (CA), Grimsby 1995 (GR), Farmington Hills 2002 (FH), Sakai 2003 (SA), Hunter 2004 (HT), Yerseke 2006 (YE), Den Haag 2006 (DH), Osaka 2007 (OS), Apeldoorn 2008 (AP), New Orleans 2009 (NO), Sydney 2012 (SY), San Francisco 2017 (SF), Hong Kong 2019 (HK). The negative control (–) corresponds to wells without antibody.

The first major GII.4 norovirus variant, Grimsby 1995 (also known as US95/96), was identified in the mid-1990s and was responsible for widespread outbreaks in multiple countries^16, 17^. This was followed by the emergence of Farmington Hills 2002, and subsequently, new variants appeared approximately every 2–3 years, including Hunter 2004, Sakai 2003, Yerseke 2006, Den Haag 2006, Osaka 2007, New Orleans 2009, and Sydney 2012^18, 19^. Contrary to the established pattern of variant emergence, Sydney 2012 has remained the predominant GII.4 virus. The antigenic variation among these variants has been linked to changes in several antigenic sites (A, C, D, E, and G) located on the outermost region of the VP1 protein (Figure 1a), particularly near the histo-blood group antigen (HBGA) binding sites^13, 14, 15, 20, 21, 22^. HBGAs are a diverse group of carbohydrates expressed on the surface of host cells and serve as attachment factors that facilitate norovirus entry. Importantly, antibodies that block the interaction between VP1 and HBGAs have been shown to be associated with virus neutralization and protective immunity^23, 24, 25, 26, 27^.

While all variable antigenic sites contribute to the diversification of GII.4 variants^12, 13, 15, 25^, antigenic site A was the primary target of the immune response in animals immunized with multiple variants such as Farmington Hills 2002, Hunter 2004, Yerseke 2006, and Den Haag 2006^22, 24^. However, the immunodominance of site G gradually increased over time, and by the emergence of the Sydney 2012 variant, both sites A and G shared the majority of the antibody response^24^. Interestingly, in animals immunized with site G-depleted Sydney 2012 virus-like particles (VLPs), the presence of antibodies targeting antigenic site G appeared to restrict the HBGA-blocking activity of antibodies directed against antigenic site A^24^. While the epitope immunodominance for each variant can be affected by previous infections^28, 29^, a similar pattern of epitope immunodominance has been described in humans, i.e. a stronger immunodominance from antigenic site A was detected in archival strains, and a more balanced immunodominance of sites A and G in Sydney 2012 infected individuals^28, 29^. Thus, a better understanding of the immune responses elicited to the immunodominant antigenic sites A and G could be critical for GII.4 vaccine development.

In this study, we analyzed a large panel of mouse monoclonal antibodies (mAbs) generated against the Sydney 2012 variant to investigate the genetic diversity of the antibody response elicited by this pandemic variant. Structural analysis of two representative mAbs, mapping to antigenic sites A and G, revealed that the immune response to Sydney 2012 viruses is predominantly directed toward epitopes spanning both antigenic sites. The structural positioning of antigenic site G appears to play a key role in shaping neutralizing immune responses. The information provided here could inform the rational design of cross-protective vaccines against GII.4 noroviruses.

## 2. Results

### 2.1. Genetic characterization of antibodies mapping to antigenic sites A and G.

Previously, we generated a panel of mouse mAbs that targeted multiple epitopes on the VP1 protein from the Sydney 2012 strain^24^ (Figure 1a). To assess genetic features of antigenic site-specific recognition and breadth, we sequenced the variable region (Fab) of these antibodies. Out of 44 antibodies, we successfully identified paired heavy and light chain sequences for 40 mAbs. The heavy chain sequences showed a bias toward the IGHV1 germline across all epitopes (27/40), whereas the light chain repertoire used a more diverse set of variable genes (Figure 1b, Figure S1). Nearly all mAbs recognizing epitopes in sites A and G used IGHV1 and IGKV4 genes and exhibited the longest CDRH3 regions, suggesting genetic requirements for binding this immunodominant site (Figure 1b–c). Notably, except for 26F10, these A and G antibodies clustered together phylogenetically and separated from mAbs targeting only A or G epitopes (Figure 1d, Figure S1). One antibody, 29A9, initially classified as binding only to antigenic site G^24^, belongs to the same clonotype as 13E9, which binds A and G. These mAbs differ by only 1 amino acid at position 107 in the CDRH3, an isoleucine in 13E9 and an alanine in 29A9 resulting from 2 nucleotide substitutions (Table S1). Similarly, antibodies 25H7 and 24A12, classified as binding on antigenic site G and additional sites including E^24^, clustered genetically with A and G antibodies based on their CDRH3 sequences (Figure 1d). Phylogenetic analysis does not completely explain the differences in antibody breadth, as there are multiple binding patterns observed across 27 GII.4 viruses, regardless of epitope (Figure 1d–e). The greatest deviation in binding across the panel can be attributed to viruses from Sakai 2003, Den Haag 2006 variant, and Osaka 2007 variants (Figure 1e).

To better understand how mechanisms of immunodominant site recognition affect antibody breadth and function, we next sought to solve the structures of two neutralizing antibodies targeting different antigenic sites. First, we chose the antigenic site G-targeting mAb 17A5, as it retains strong binding to the Gabon561/2018 (SF) and HongKong/2019 (HK) viruses, representing the newly detected San Francisco 2017 and Hong Kong 2019 variants, respectively, and has a notably short CDRH3 (9 aa) (Figure 1 and Table S1). Second, we chose 24C10 as the prototypical AG mAb to study, as it shared variant binding patterns with several other AG mAbs but is distinct from the activity of 17A5 (Figure 1e). Both antibodies exhibited moderate to high affinity, with K_D_ values of 97.7 nM and 4.1 nM for 17A5 and 24C10, respectively (Figure S2a).

### 2.2. High-resolution structures of antibodies 17A5 and 24C10 demonstrate binding to multiple antigenic sites and different HBGA-blocking mechanisms

The crystal structures of antibodies 17A5 and 24C10 in complex with the norovirus VP1 P domain were resolved at resolutions of 3.36 Å and 2.7 Å, respectively (Table S2). We note that AlphaFold 3 could not predict either structure accurately. Confidence scores for 24C10 yielded a pTM of 0.47 and ipTM of 0.34, while those of 17A5 yielded a pTM of 0.51 and ipTM of 0.39, all below the 0.6 threshold considered indicative of a reliable prediction (Figure S3). This highlights the continued difficulty of accurately modeling antibody-epitope interactions computationally.

**Figure 2:**
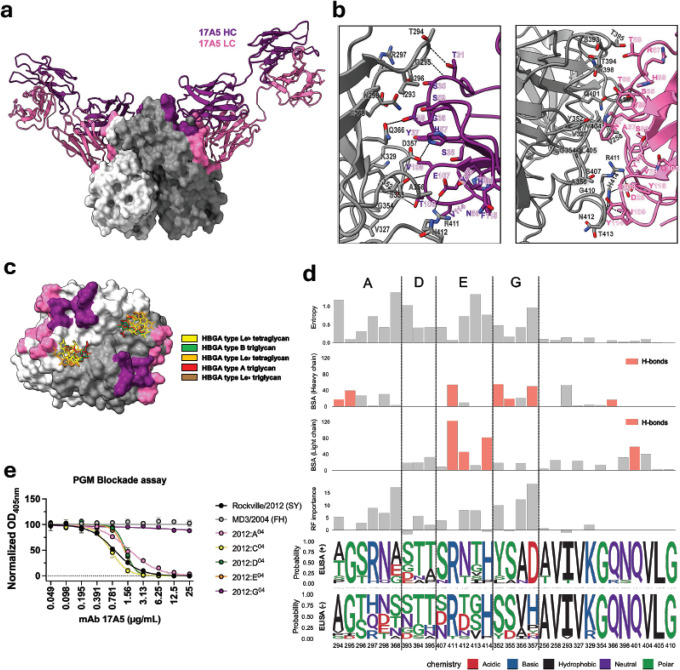
Crystal structure and functional characterization of Fab 17A5 bound to GII.4 Sydney 2012 VP1 P domain. **a,** Side view of neutralizing Fab 17A5 in complex with the recombinant GII.4 RockvilleD1/2012/USA VP1 P domain dimer. The Fab is shown in ribbon representation and the P domain dimer in surface representation. Heavy and light chains are colored purple and pink, respectively; VP1 P domain protomers are shown in light and dark gray. 17A5 binds laterally to VP1, with both chains wrapping along the side surfaces of the two protomers. **b,** Close-up of the Fab–P domain interface showing heavy-chain (left) and light-chain (right) interactions. Antibody residues are numbered in IMGT numbering format. VP1 contact residues are indicated. Hydrogen bonds are shown in black dashes. **c,** Surface representation of the P domain dimer highlighting the 17A5 epitope (heavy chain, purple; light chain, pink) superimposed with previously defined HBGA carbohydrate binding sites (PDB: 4OPO, 4OP7, 4WZE, 4WZT, 4X0C). **d,** Structural and evolutionary features of VP1 residues comprising the 17A5 epitope. Shannon entropy values were calculated using a GII.4 dataset (n = 3,145)^35^. Buried surface area (BSA) was determined using PDBePISA. Residue importance (Mean Decrease Accuracy from random forest analysis) reflects predicted contribution to antibody binding specificity (see Fig. 1e). Logo plots show amino acid distributions among ELISA-positive and -negative viruses. Residues are grouped by antigenic site; shaded residues form hydrogen bonds with the antibody. **e,** HBGA blockade curves for homologous (RockvilleD1/2012) and heterologous (MD3/2004) VLPs, including antigenic site–swapped mutants. The graph indicates best-fit non-linear regression lines and mean and standard deviation of normalized OD_405nm_ values measured in duplicate wells.

The ~985 Å^2^ epitope recognized by antibody 17A5 encompasses 30 residues on VP1 and involves interactions with both the heavy and light chains (Figure 2a–b). The 17A5 epitope does not directly overlap with residues involved in HBGA binding (Figure 2c); however, the interacting residues span antigenic sites A, D, E, and G and include several conserved positions (12 of 30) adjacent to variable residues (Figure 2b and d). The heavy chain interacts with residues from antigenic sites A, G, and E, whereas the light chain primarily engages those from antigenic site E, and both chains contact conserved residues (Figure 2d). To further support the structural analyses and cross-reactivity patterns observed (Figure 1e), we used random forest algorithms to identify residues that play a major role in antibody binding selectivity across the panel of GII.4 viruses. The five most important residues explaining the binding pattern of 17A5 included three amino acids from antigenic site G (352, 356, and 357), one from site A (368), and one from site E (412). Notably, only the residues from antigenic site G were highly conserved among viruses showing positive binding (Figure 2d and Figure S4). The two viruses representing the newer variants, Gabon561/2018 (SF) and HongKong/2019 (HK), exhibited one and three mutations, respectively, in antigenic site G compared with the Rockville/2012 (SY) virus. Importantly, both retained aspartic acid at position 357, which is predicted to be critical for binding, thereby explaining the reactivity of 17A5 with these variants. The reactivity with the Oregon/2012 (DH) virus, the only Den Haag 2006 variant virus with aspartic acid at position 357 (Figure S4), further supports these observations. To experimentally validate these bioinformatic and structural predictions, we tested VLPs containing mutations in each of these antigenic sites. Only mutations in antigenic site G resulted in a loss of HBGA blocking (Figure 2e), confirming the central role of antigenic site G in 17A5 binding.

**Figure 3:**
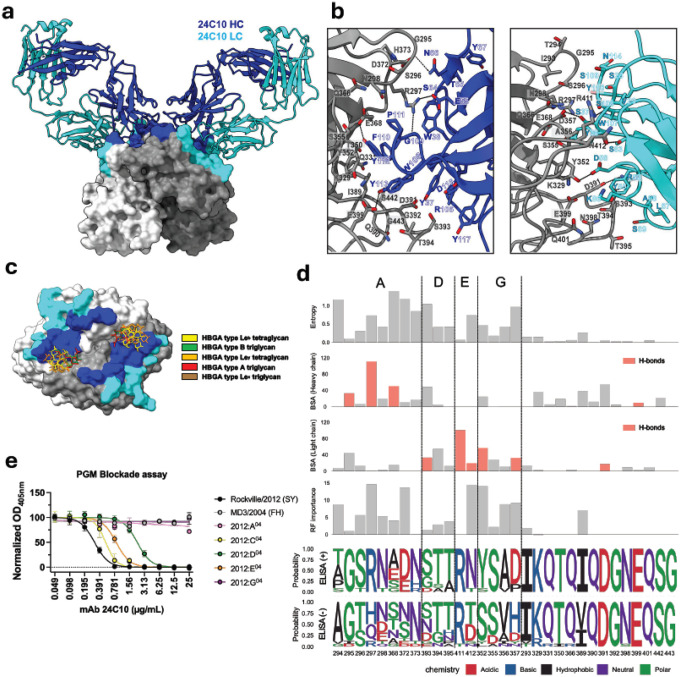
Crystal structure and functional characterization of Fab 24C10 bound to GII.4 Sydney 2012 VP1 P domain. **a,** Side view of neutralizing Fab 24C10 bound to the GII.4 RockvilleD1/2012/USA VP1 P domain dimer. Structural representations are as in Fig. 2. Heavy and light chains are shown in blue and cyan, respectively. 24C10 engages the apex of the P domain dimer. **b,** Close-up of the Fab–P domain interface showing heavy-chain (left) and light-chain (right) interactions. Antibody residues are numbered in IMGT numbering format. Hydrogen bonds are shown in black dashes. **c,** Surface representation of the P domain dimer highlighting the 24C10 epitope (heavy chain, blue; light chain, cyan). The heavy chain sterically overlaps with the HBGA carbohydrate binding site. **d,** Structural and evolutionary features of VP1 residues comprising the 24C10 epitope, analyzed as described in Fig. 2d. **e,** HBGA blockade curves for homologous (RockvilleD1/2012) and heterologous (MD3/2004) VLPs, including antigenic site–swapped mutants. The best-fit non-linear regression lines with mean and standard deviation of normalized OD_405nm_ values measured in duplicate wells are presented.

The epitope recognized by antibody 24C10 is different from that of 17A5 (Figure 3a–b) and overlaps with the HBGA binding sites (Figure 3c), explaining its strong blocking activity (EC_50_ = 0.2022 [95% CI 0.1867–0.2213] μg/mL) as compared to 17A5 (EC_50_ = 0.3918 [95% CI 0.3387–0.4527] μg/mL). Interestingly, the 17A5 Fab displayed a decreased blockade potency compared to its mAb form, while the blockade potency of the 24C10 antibody was unaffected (Figure S2b-c), supporting structural observations suggesting direct blockade by 24C10 and indirect steric blockade by 17A5. The ~1004 Å^2^ epitope recognized by 24C10 encompasses 31 residues on VP1 (Figure 3d). These residues span antigenic sites A, D, E, and G and include 14 additional conserved positions (Figure 3d). Interaction with residues from antigenic sites A and G are mediated by H-bonds, hydrophobic interactions, and an electrostatic interaction (297) with the heavy and light chains, and two residues from antigenic site E (411, 412) form H-bonds with the light chain (Figure 3b and d). Although residue 329 is highly conserved among most variants (Figure 3d), it presents an arginine substitution in the Farmington Hills 2002 and Hunter 2004 variants that circulated between 2002 and 2008 (Figure S5a and b). Residue 329 is structurally adjacent to residues 352 and 368, which map to antigenic sites G and A, respectively, suggesting that the local composition of these immunodominant antigenic sites plays an important role in shaping antibody recognition (Figure S5b). Random forest analyses predicted that the top ten residues explaining the binding patterns from 24C10 against the panel of GII.4 viruses map to antigenic site A (n=5; 294, 296, 297, 298, 372), G (n=3; 352, 356, 357), E (n=1; 412), and one conserved residue 389. Conservation analyses from those 10 residues further explains binding patterns with the panel of GII.4 viruses (Figure 1d). For example, the lack of reactivity with 16 of the 27 viruses tested can be explained by mutations at position 352 (Figure S4). The exceptions are Sakai/2005 (SA) and Gabon561/2018 (SF); however, the reactivity to Sakai/2005 (SA) can be explained by the presence of alanine at position 368, which does not seem to affect binding as other viruses carrying that mutation maintain positivity with 24C10. Moreover, although Gabon561/2018 (SF) does not present a mutation in residue 352, it does present an asparagine at residue 356 and several mutations at antigenic site A, including a histidine at position 297 (Figure S4). To further confirm the contribution of individual antigenic sites to 24C10 recognition, we evaluated VLPs containing targeted mutations in each of these antigenic sites. Alterations within antigenic sites A and G had the most pronounced effect on HBGA blocking titers (Figure 3e), indicating a major role for these sites in mediating antibody function. Mutations in antigenic site D, and to a lower degree in E and C, also demonstrate a role in antibody evasion (Figure 3e).

### 2.3. Sequence and structural interactions of 24C10 provide insights on the antigenic evolution of GII.4 noroviruses

**Figure 4:**
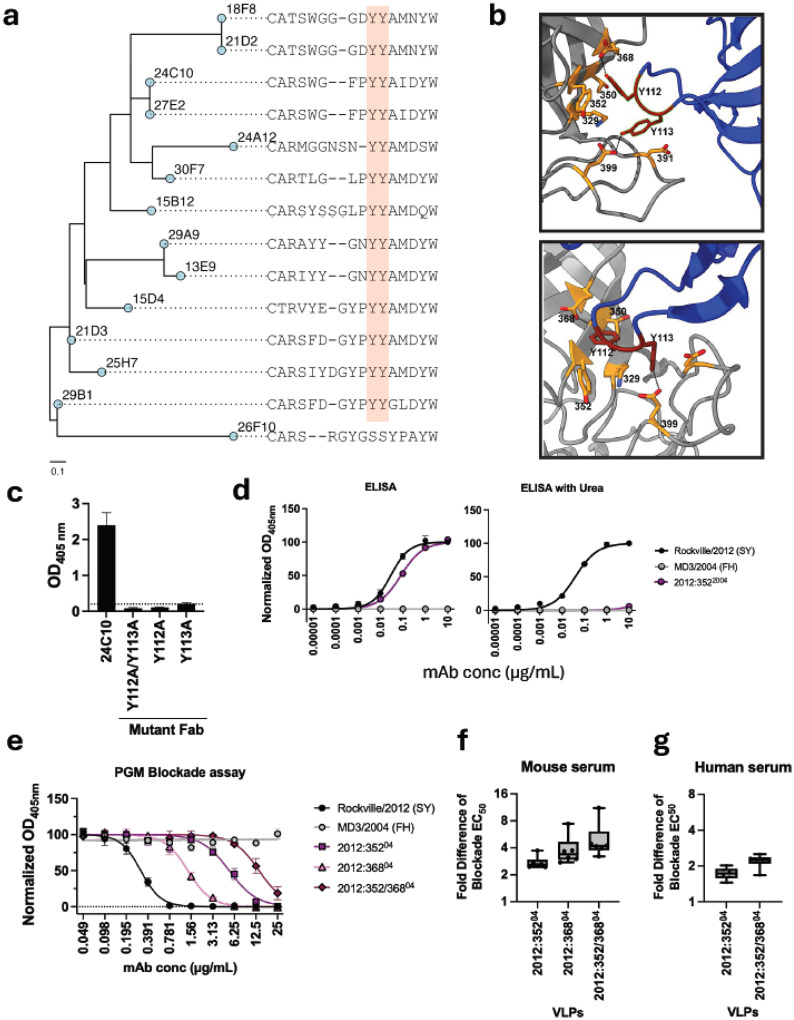
A characteristic motif in the CDRH3 from AG antibodies explain their fondest to AG epitopes. **a,** Genetic relationships based on amino acid sequences from the CDR3 of the heavy chain of antibodies mapping to epitopes encompassing antigenic sites A and G. **b,** Close-up of the 24C10–P domain interface showing the two tyrosines motifs (red) and the five residues on VP1 (orange) interacting with these tyrosines. **c,** Mutations in tyrosines 112 and 113 abrogates Fab binding to RockvilleD1/2012/USA VLPs. Fabs were tested at 10 μg/mL and VLPs at 0.5 μg/mL. Mean and standard deviation from duplicates are presented. The horizontal dashed line indicates OD_405nm_ = 0.2. **d,** Reactivity of 24C10 mAb (starting concentration: 10 μg/mL) against homologous (RockvilleD1/2012) and heterologous (MD3/2004) wild-type VLPs (0.5 μg/mL), and RockvilleD1/2012 mutant 352 VLPs under normal and chaotropic (Urea 7M) conditions. **e,** HBGA blocking activity of 24C10 mAb (starting concentration: 25 μg/mL) against homologous, heterologous wild-type VLPs, as well as single- and double-mutant (Y352S and E368N) VLPs. **f-g,** EC_50_ HBGA blocking fold difference of mouse (n = 6) and human sera (n = 6) against single and double mutants (Y352S and E368N) VLPs as compared with homologous RockvilleD1/2012/USA VLPs. The line graphs in panel **d** and **e** present best-fit non-linear regression lines and mean values with standard deviation measured in duplicate wells. The boxplot in panel **f** and **g** present individual fold difference of EC_50_ titers with whiskers showing minimum and maximum values.

Given the prominent role of AG-targeting antibodies (24C10-like) in shaping immunodominance during the emergence of GII.4 variants, we next examined the sequence characteristics of all antibodies mapping to AG epitopes, including those genetically clustered with this group (25H7, 24A12 and 29A9). Notably, the CDRH3 of all but one antibody (26F10) present two tyrosines (Y112, Y113) that interact with six residue sidechains (329, 350, 352, 368, 391, 399) on the norovirus VP1 (Figure 4a–b). Since the substitution patterns of residues 352 and 368, correlates with variant circulation (Figure S5a and c), we speculated that these two tyrosines are key for antibodies targeting the 24C10 epitope. Therefore, we created Fabs from three representative antibodies (24C10, 29B1, and 29A9) with alanine substitutions at the two conserved tyrosines, i.e. Y112A and Y113A. We observed a complete abrogation of binding with either single or double mutations (Figure 4c, Figure S6), confirming the essential role of this two-tyrosine motif in AG antibody binding. We then noted that a conserved substitution (Y352S) in VP1 differentiated viruses reactive to 24C10 from those that were non-reactive. (Figure 1e and Figure S4). We first tested whether this single mutation on 352 affected the binding of the AG antibodies in our panel. We noticed that this mutation has little effect on the binding of 24C10, however, when exposing to a chaotropic agent (Urea 7M) reactivity is completely abrogated in Y352S mutant Sydney 2012 VLP but not in wild-type VLPs (Figure 4d), suggesting that antibody affinity and/or avidity is affected. Moreover, when testing the HBGA blocking of 24C10 against the Y352S mutant Sydney 2012 VLP, the EC_50_ (measured as antibody concentration, μg/mL) increased ≥16-fold. We next tested the role of E368N mutation of VP1 on HBGA blocking activity of 24C10 and observed a ~4-fold less potent EC_50_ compared with the wild-type VLPs. The double mutant (Y352S, E368N) demonstrated an additive effect, further reducing the blockade potency (Figure 4e). Of note, these mutations had a differential impact on blocking of other AG antibodies. For example, single mutations had limited impact on 29B1 blocking activity, whereas the double mutation acted synergistically to reduce 29B1 blocking activity (Figure S6). In contrast, the single mutation Y352S had a strong impact on 29A9 blocking activity (Figure S6), consistent with our initial observation that mutations in antigenic site G – not site A – determine binding of this antibody^24^. Based on the remarkable role of Y352S and E368N mutations on the emergence of new viruses (Figure S5c) and binding of the immunodominant AG antibodies, we then tested the role of these two mutations on reducing the HBGA blocking activity of AG antibodies as part of the polyclonal response. In animals immunized with GII.4 Sydney 2012 VLPs, the presence of mutations in residues 352 and/or 368 reduced HBGA blocking EC_50_ titers by >2-folds (Figure 4f). Moreover, serum from humans infected with GII.4 norovirus exhibited a ~2-fold reduction in HBGA-blocking titers against the mutant VLPs compared with wild-type Sydney 2012 VLPs (Figure 4g and Figure S7), further confirming the critical role of these two residues in shaping immune responses to – and driving the antigenic evolution of – GII.4 noroviruses.

## 3. Discussion

Despite the substantial societal burden imposed by norovirus infections, no vaccine or antiviral is currently available to control norovirus disease. Because norovirus genotype GII.4 plays a leading role in causing acute gastroenteritis worldwide, all vaccine candidates under development include this genotype in their formulation. One of the major obstacles to the development of cross-protective vaccines is the continuous emergence of GII.4 variants, which facilitates reinfection through escape from antibodies elicited by prior variants. Significant efforts have been made to identify and characterize antibodies that target conserved regions of the norovirus capsid protein and to define their atomic interactions; however, such epitopes appear to be weakly immunogenic or are detected in only a small proportion of naturally infected individuals^24, 30^. In contrast, the most immunodominant epitopes that elicit neutralizing responses map to the apex of the capsid protein, which also represents the most variable region of VP1^31^. Despite the variability of this region, broadly reactive antibodies have been described in individuals and experimental animals^31, 32, 33^; thus, a better understanding of how immunity is elicited to these epitopes could provide critical information for vaccine design.

In this study, we defined the genetic and structural features of antibodies that target two of the most immunodominant antigenic sites of the GII.4 norovirus capsid and demonstrate that: (i) their epitopes span residues from multiple variable antigenic sites, yet antigenic site A and/or G play the dominant role in immune escape; (ii) these epitopes also encompass several conserved residues that, under specific conditions (e.g., the R329K substitution), can mutate and provide additional pathways for immune escape; and (iii) at least two distinct epitopes involving antigenic site G contribute to immune responses against GII.4 noroviruses.

High-resolution analysis of the interactions between antibodies 24C10 and 17A5 and the GII.4 norovirus VP1 reveals that these two G-site–mapping antibodies recognize distinct epitopes and engage VP1 at different angles of attack. Antibody 17A5 binds laterally to the VP1 dimer and does not directly contact residues involved in HBGA binding; instead, its ability to block VP1–HBGA interactions is likely mediated by indirect steric hindrance resulting from the Fc region (Figure S2). In contrast, the epitope recognized by antibody 24C10 overlaps with the HBGA-binding site, providing a mechanistic explanation for its stronger blocking activity compared with 17A5. The structural data also explains their reactivity patterns and confirms the important inter-relationship of several variable antigenic sites in eliciting neutralizing antibodies^24, 25, 33^. This concept is particularly relevant to the central role of site G in the antigenic diversification of GII.4 noroviruses during 2002 – 2012. Despite its involvement in immune responses against the Grimsby 1995 variant, residues mapping to antigenic site G remained conserved at that time^15^. Throughout the diversification of GII.4 viruses, variability within antigenic site G began with substitutions at residue 355 (S355D) in the Farmington Hills 2002 variant^34^. Although antigenic site G does not appear to be a dominant target of immunity to the Farmington Hills 2002 variant, a substantial number of antibodies targeting antigenic sites G and E were detected in experimentally immunized animals^24^. Thus, it is possible that antibodies similar to 17A5, which also recognize several residues from antigenic site E (n=5), were more frequently elicited by the Farmington Hills 2002 variant. Over the subsequent evolution of GII.4 variants, antibodies resembling 24C10, which target residues from antigenic sites A and G, may have become more prevalent and exerted increasing immune pressure on these positions, particularly residues 352 and 368. The observation that 24C10-like antibodies map to multiple antigenic sites – namely A, G, D, and E (Figure 3) – supports previous bioinformatic predictions indicating that synchronous changes across residues from distinct antigenic sites^12, 24^ are associated with the emergence of GII.4 variants. While this framework explains the rapid turnover of variants during 2002 – 2012, it does not fully explain the lack of antigenic evolution in Sydney 2012^35^ or its prolonged predominance. We speculate that the pressure exerted by 24C10-like antibodies to four antigenic sites constrains antigenic diversification by competing with other functional roles of VP1, such as binding to HBGA attachment factors or the yet unidentified cellular receptor, thereby locking the antigenic evolution of Sydney 2012 viruses. Finally, structural analyses of 17A5 provides a mechanistic explanation for its binding to two recently emerging variants, San Francisco 2017 and Hong Kong 2019. Most of the variability of these two variants relative to Sydney 2012 was associated with residues in antigenic site A, rather than site G, which is the primary interacting site for 17A5-like antibodies (Figure S4).

The prevalence of tyrosine residues within antibody CDR regions involved in antigen binding has been demonstrated for antigens of diverse nature^36, 37^. Accordingly, the specific motif comprising two conserved tyrosines in the immunodominant 24C10-like antibodies is of particular interest and should be considered in studies of immune responses in individuals infected with GII.4 noroviruses. Notably, these two tyrosines interact with residues 352 and 368, which play key roles in the antigenic evolution of this genotype. In addition, these tyrosines also interact with residue 329, which is often mutated in the Farmington Hills 2002 variant. This observation is highly relevant to the study of viral evolution, as residues initially considered conserved can acquire mutations that may create additional pathways for viral immune escape.

The genetic and structural analyses described in this study provide a mechanistic explanation for the diversification and emergence of GII.4 variants during the 2000s and the central role of antigenic site G-mapping antibodies in the immune responses elicited to other variable antigenic sites. Indeed, the presence of antibodies against antigenic site G were shown to restrict the blocking activity, and elicitation, of antibodies targeting site A^24^. This information could be critical for the development of effective vaccines as the presence of broadly reactive, and blocking, antibodies mapping to antigenic site A was demonstrated in infected individuals and immunized animals. Overall, the data presented here should help in the rational design of vaccine candidates to this ever-changing norovirus genotype.

## 4. Materials and Methods

### GII.4 norovirus VLPs production:

The VP1 proteins of representative GII.4 viruses/variants (Table S3) were expressed using the Bac-to-Bac baculovirus expression system (Gibco) in *Spodoptera frugiperda* (Sf9) insect cells (ATCC, CRL-1711), as previously described^12^. Briefly, expressed VP1 proteins self-assembled into VLPs, which were purified by ultracentrifugation through a 25% (w/v) sucrose cushion (4 h at 98,400 × g, 4 °C), followed by cesium chloride density gradient centrifugation (18 h at 280,000 × g, 15 °C). Purified VLPs were dialyzed against 1× PBS (pH 7.4; Gibco) using Slide-A-Lyzer dialysis cassettes (Thermo Scientific). Norovirus VP1 expression was confirmed by Western blot using a mouse anti-norovirus monoclonal antibody (30A11; 1:10,000 dilution)^38^ and a horseradish peroxidase (HRP)-conjugated goat anti-mouse IgG secondary antibody (SeraCare, Cat# 5220–0339; 1:2,000 dilution). VLP integrity was confirmed by transmission electron microscopy following negative staining with uranyl acetate. VLP concentrations were quantified using the Qubit Protein Assay Kit (Invitrogen). In addition, mutant VP1 constructs were generated to exchange either entire antigenic sites^24^ or individual residues within antigenic sites A and G (positions 352, 368, or both) between the GII.4 Sydney 2012 and Farmington Hills 2002 variants. Mutant VP1 sequences were synthesized and expressed as VLPs using the same procedures described above.

### Antibody development and sequencing:

Antibodies used in this study were generated in previous study^24^. Briefly, Balb/c mice were immunized with with GII.4 RockvilleD1/2012 VLPs three times followed by purification of splenic B cells for hybridoma fusion (Genscript). Monoclonal hybridomas secreting VLP-specific IgG antibody were down-selected for further study. Sequencing using mouse-specific IGH and IGK/L primers generated paired heavy and light chain sequences for 40 antibodies described in this study (Genscript).

### Antibody sequence analysis:

Antibody sequences generated from GII.4 RockvilleD1/2012 hybridomas were aligned and annotated using IMGT numbering^39^. IMGT/V-quest was used to align IGH and IGK sequences to infer V-, D- and J-gene identity, and acquired nucleotide mutations^39^. IMGT/DomainGapAlign was used to align and assess acquired amino acid mutations. Clonotypes were defined as antibody sequences sharing identical Variable genes, have the same CDR3 length and share >70% identity in their CDR3 sequences. Phylogenetic trees were constructed using amino acid sequences and maximum-likelihood method as implemented in MEGA v11^40^ and visualized using ggtree package v3.16.0 in R v4.5.2.

### Fab expression and purification:

The heavy and light chain variable region sequences from antibodies 24C10, 17A5, 29B1, and 29A9, were synthesized and cloned into pTwist CMV BG WPRE Neo for expression (Twist Biosciences). The heavy chain plasmid was synthesized with a C-terminal Streptag II for purification. Equal masses of heavy and light chain plasmid DNA were mixed with Expifectamine 293 reagent and used to transfect Expi293 F cells (Thermo Fisher). Expi293 F cells were incubated with gentle rotation for 5–7 days to allow for ample protein expression. Cells were then harvested by centrifugation and supernatant transferred to a new sterile container. Fab was purified from the supernatant using Streptactin XT resin (IBA Bioscience). Purified Fab was eluted from the resin, buffer exchanged into 1X PBS using a 10KDa MWCO concentrator and filtered using a 0.22 μM filter (Millipore Sigma). Fab purity was confirmed using SDS-PAGE, and functional binding by ELISA with the GII.4 RockvilleD1/2012 VLPs.

### Animal serum samples:

Hyperimmune mouse sera against the wild-type GII.4 RockvilleD1/2012 VLPs were produced in Balb/c mice following the same protocol indicated above and elsewhere^12, 24^. This animal study was approved by the FDA Institutional Animal Care and Use Committee, protocol number 2018–41. Hyperimmune guinea pig sera, used for the detection of norovirus VLPs in the HBGA-binding and blockade assays, were produced as previously described^23^, under the approved animal protocol number 2017–29.

### Human serum and plasma samples:

Human serum and plasma samples were obtained from three different studies. Three samples from NIH Blood Bank were collected in January 2025 from healthy American volunteer donors at age ≥18 years old (ClinicalTrials.gov ID: NCT00001846). One sample was collected in March 2024 from a healthy American individual with a known history of GII.4 Sydney infection. Two samples from Peruvian children known infected with GII.4 Sydney virus were collected in July or August 2017 as part of a previous birth cohort study conducted in Lima, Peru. The use of human samples was approved by the institutional review board ([IRB], protocol number CBER IRB 16–069B).

### Enzyme-linked immunosorbent assay:

To determine the binding patterns of mAbs and Fabs against different GII.4 variants and mutant VLPs, VLPs (0.5 μg/mL in 1×PBS, pH 7.4) was coated on the 96-well “U” bottom microtiter plates (Thermo Scientific) overnight at 4°C and blocked for 1 hour at room temperature with 5% blocking buffer (Bio-Rad). The mAbs (10 μg/mL or 10-fold serial dilution of 10 μg/mL) were incubated on the VLPs-coated plates for 2 hours at room temperature. After three washes with 1×PBS, 0.1% Tween 20, plates were incubated with 1:2,000 dilution of HRP-conjugated goat anti-mouse IgG secondary antibody (SeraCare, Cat#5220–0342) for 1 hour at room temperature and washed three times. Binding intensity was visualized using ABTS 1-Component Microwell Peroxidase Substrate Kit (SeraCare). The OD_405nm_ values were measured using SPECTROstar Nano plate reader (BMG LABTECH). The measured OD_405nm_ values were plotted as a heat map or normalized and plotted as a line graph using GraphPad Prism v10. To remove binding signals coming from weak interactions between mAbs and VLPs, mAbs:VLPs complex on the plate was exposed to a chaotropic agent (Urea 7M) for 10 min at room temperature after the incubation step of mAbs and a single wash. After the exposure to urea, binding signals of mAbs were similarly detected as described above.

### Biolayer Interferometry (BLI) assay:

The binding kinetics of monoclonal antibodies 24C10 and 17A5 to norovirus VP1 P domain were evaluated using BLI on an Octet RED384 instrument using the Data Acquisition Software (version 11.1.1.19). All experiments were conducted at 25°C using 1X Octet^®^ Kinetics Buffer (Sartorius) and shaking at 1,000 rpm. Streptavidin (SA) biosensors were hydrated in Octet^®^ Kinetics Buffer for a minimum of 30 minutes before use. The binding assay protocol consisted of five sequential steps: (1) an initial 60-second baseline equilibration in assay buffer; (2) 180 second loading phase with Avi-tag-biotinylated norovirus VP1 P domain at 5 μg/mL; (3) 60-second baseline in assay buffer; (4) a 300 second association phase with serially diluted monoclonal antibody Fab (concentration range: 25–200 nM); and (5) a 600-second dissociation phase in assay buffer. Traces were reference subtracted with a no antibody control, aligned to the baseline, and fit using 1:1 model of binding. The traces were fit globally for curves in each dilution series. Average K_D_ values are reported as the mean and standard deviation of three independent experiments.

### HBGA-blockade assay:

To determine the blockade activity of antibodies against different GII.4 VLPs, the HBGA-blockade assays were conducted using pig gastric mucin III (PGM III, Sigma-Aldrich) as a source of HBGA carbohydrates. Briefly, PGM III (10 μg/mL in 1×PBS, pH 7.4) was coated on 96-well “U” bottom vinyl plates (Costar) overnight at 4°C and blocked for 1 hour at room temperature with 5% blocking buffer (Bio-Rad). Another 96-well “U” bottom vinyl plate was blocked overnight at 4°C and used to incubate duplicates of 2-fold serial dilutions of antibodies (starting from 100 μg/mL or 25 μg/mL) or serum (starting from 1:100 or 1:50 dilution) in 5% blocking buffer with GII.4 VLPs (0.25–0.5 μg/ml, depending on affinity of VLPs to the PGM III) for 1 hour at 37°C. Pre-incubated mAb-VLPs mixture was then added to PGM III-coated plates and incubated for additional 1 hour at 37°C. A 1:5,000 dilution of pooled serum from guinea pigs immunized with Sydney 2012 and Farmington Hills 2002 variant VLPs (RockvilleD1/2012 and MD3/2004) were used as primary detection antibodies against bound VLPs. Binding signals of VLPs on PGM III was determined using goat anti-guinea pig IgG-HRP (SeraCare, Cat#5220–0366, 1:2,000 dilution) and ABTS (SeraCare). The OD_405nm_ values of mAb or serum dilutions were measured using SPECTROstar Nano plate reader (BMG LABTECH). The EC_50_ values, measured by reciprocal of serum dilution, were calculated from the normalized OD_405nm_ curves using GraphPad Prism v10.

### Expression and purification of recombinant norovirus capsid P domain for crystal structure determination:

A codon-optimized synthetic gene encoding the P domain sequence of the major capsid protein VP1 of wild-type norovirus GII.4 (RockvilleD1/USA/2012, Accession No: KY424328, amino acids 221–530) was cloned into the pET28a-6xHis-MBP-TEV vector downstream of the N-terminal 6xHis-tag, maltose-binding protein (MBP), and TEV protease cleavage site (GenScript)^24^. The plasmid was transformed into *E. coli* strain T7 Express (New England Biolabs) and grown at 37°C until the OD_600nm_ reached 0.6. Protein expression was induced with 1 mM isopropyl β-D-thiogalactopyranoside (IPTG) at 18°C for 18 h. Cells were harvested by centrifugation and lysed by sonication in lysis buffer (20 mM Tris-HCl pH 8.0, 300 mM NaCl, 20 mM imidazole) supplemented with 2 mM MgCl_2_, 0.0125 U/ml Benzonase (Millipore), and EDTA-free protease inhibitor cocktail (Millipore 539137). The cell lysate was clarified by centrifugation at 40,000g for 30 min, filtered through a 0.2 μm Acrodisc filter (Pall), and the P domain protein was purified using TALON metal affinity chromatography. The protein was digested overnight with TEV protease to remove the 6xHis-MBP and dialyzed into TBS buffer containing 20 mM Tris-HCl pH 8.0 and 150 mM NaCl. The cleaved P domain was separated from the 6xHis-MBP using metal affinity chromatography, concentrated to 7.0 mg/ml, and purity was assessed by SDS-PAGE with Coomassie Blue staining.

### Expression and purification of recombinant monoclonal antibody Fabs 24C10 and 17A5 for crystal structure determination:

The protein-coding sequence of antibodies 24C10 and 17A5 heavy and light chains were determined and chemically synthesized (Twist Biosciences). The heavy chain antibody Fab sequences were cloned into pcDNA3.4 plasmids in-frame with an N-terminal signal peptide and C-terminal 6xHis-tag, while light chain antibody Fab sequences were cloned in-frame with the signal peptide only. CHO-S cells (8×10^7^ cells) were co-transfected with 60 μg each of heavy and light chain plasmids using MaxCyte electroporation with an OC-400 cuvette. Transfected cells were cultured in CD-OptiCHO medium (Gibco #12681029) and fed daily with CHO CD EfficientFeed A (Gibco #A1023401) supplemented with 7 mM L-glutamine, 5.5% glucose, and 23.4 g/L yeastolate. At 24 h post-transfection, 1 mM sodium butyrate was added, and cultures were maintained at 32°C, 8% CO_2_, 85% humidity, 135 rpm for 7 days. Culture supernatants were harvested by centrifugation, supplemented with EDTA-free protease inhibitor cocktail (Millipore 539137) and filtered through a 0.2 μm filter. The clarified supernatant was diluted 1:1 (v/v) with binding buffer (20 mM sodium phosphate pH 7.4, 500 mM NaCl, 20 mM imidazole) and loaded onto a HisTrap Excel column (Cytiva) using an ÄKTA purification system. After washing with binding buffer, bound Fab proteins were eluted using a linear gradient to 500 mM imidazole in the same buffer. Purified Fab was dialyzed into TBS (20 mM Tris-HCl pH 8.0, 150 mM NaCl) overnight at 4°C. Protein purity was assessed by SDS-PAGE with Coomassie Blue staining.

### X-ray crystallography structure determination of norovirus capsid P domain – Fab complexes:

Affinity-purified norovirus P domain was incubated with 2–3-fold molar excess Fab overnight at 4°C and the resulting complex was purified by size-exclusion chromatography on a Superdex 200 10/300 column. Peak fractions corresponding to the P domain – Fab complex were identified by comparison with gel filtration standards and confirmed by SDS-PAGE analysis (Figure S8). The complex was concentrated to 6–7 mg/ml in TBS pH 8.0 and crystallized using the hanging-drop vapor diffusion method at 22°C. Crystals were grown in 2 μl drops containing a 1:1 ratio of protein solution to reservoir solution for the 17A5 complex (0.1 M sodium acetate pH 4.6, 2 M sodium formate) or for the 24C10 complex (0.2 M HEPES pH 7.5, 8% (w/v) PEG 8000, 8% (v/v) ethylene glycol). A single crystal was transferred into a cryoprotectant solution consisting of well solution and 25% glycerol. All crystals were flash-frozen in liquid nitrogen and diffraction data from a single crystal were collected at cryogenic temperature using a wavelength of 0.97 Å at the Advanced Light Source beamlines 5.0.1 or 5.0.3. For 17A5, data was indexed and integrated using XDS, followed by scaling and merging in Aimless with a resolution cutoff of 3.36 Å based on CC1/2 and I/σl statistics^41, 42, 43, 44^. For 24C10, data was indexed, integrated, scaled, and merged using DIALS (ccp4i23) with a resolution cutoff of 2.70 Å based on CC1/2 and I/σl statistics. A model of Norovirus P domain (PDB: 5KON) and a trimmed model of Fab generated by SWISS-model4 was used for molecular replacement with Phaser^45, 46^. Model building and refinement were performed through alternating iterations in Coot 0.9.8.76 and refined in Phenix version 1.20.1–448711^47, 48^. The final model was validated with MolProbity^49^. Data collection and refinement statistics are reported in Table 1. The model and structure factors have been deposited in the Protein Data Bank. Binding interfaces and interactions were analyzed using the PDBePISA online server (https://www.ebi.ac.uk/pdbe/pisa/). Figures and protein contact potentials were generated using ChimeraX 1.9^50^. Hydrogen bonds were defined as those having a distance of 3.2Å or less between the antibody and antigen.

### Prediction of P domain-antibody complexes:

To evaluate the performance of *in-silico* structural modeling, P domain-antibody complexes were predicted using AlphaFold 3^51^. The amino acid sequences of heavy and light chain of the antibodies 17A5 and 24C10 together with P domain sequence of the wildtype norovirus GII.4 (RockvilleD1/USA/2012) were used as inputs to Alphafold 3 to construct the prediction models of each complex. The quality of Alphafold 3 predictions were evaluated with predicted template modeling (pTM) and ipTM (Interface pTM) metrics as implemented in Alphafold 3 and by structural alignment in Chimera × 1.9 with the crystal structure obtained in this study.

### Viral Sequence Analyses and Antibody Binding Patterns Predictions:

The VP1 amino acid sequences of GII.4 variants used for ELISA were aligned using MEGA v11^40^. Amino acid sequence diversity among variants were visualized as a logo plot using ggseqlogo package v0.26^52^ in R v4.5.2. Sequence diversity at individual positions of the VP1 was calculated as Shannon entropy metrics using the Shannon Entropy-One tool as implemented in Los Alamos National Laboratory (https://www.hiv.lanl.gov/content/sequence/ENTROPY/entropy_one.html) using previously curated GII.4 VP1 sequence dataset^35^. Temporal trend of amino acid mutation(s) was summarized in R v4.5.2 and visualized in GraphPad Prism v10. Previously characterized antigenic sites were plotted on the VP1 structural model using USCF ChimeraX v1.9^50^ and PDB 7K6V. To predict residues involved in VLP:mAb interactions, we implemented random forest algorithm to predict residues of VP1 that could have contributed to the positive or negative signals in ELISA against mAbs 17A5 and 24C10. The dataset includes 27 datapoints per each mAb measured by ELISA (OD_405nm_ values) using 27 GII.4 wild-type VLPs, and the VP1 amino acid sequence information on the antibody contact residues determined by structural data. The ELISA positive group included viruses (VLPs) with ELISA OD_405nm_ values ≥0.2 and the negative included those with OD_405nm_ <0.2. Random forest-based classification was applied using randomForest package v4.7–1.2 in R v4.5.2 with 1,000 trees, which provided out-of-bag estimates of error rate = 7.41% and 11.1% in mAbs 17A5 and 24C10, respectively. The importance of individual viral residues in VLP:mAb interactions was measured as mean decrease in accuracy and visualized using ggplot2 package v3.5.2 in R v4.5.2.

## Supplementary Material

Supplementary Files

This is a list of supplementary files associated with this preprint. Click to download.

• SupplementalMaterials.pdf

## Data Availability

Structural data (coordinates and structure factors) have been deposited in Protein Data Bank (PDB accession numbers 11HA and 11HB). The raw diffraction data have been deposited at the Integrated Resource for Reproducibility in Macromolecular Crystallography 2.0 [https://www.proteindiffraction.org/].
